# Eco-Friendly Adhesives Based on the Oligomeric Condensed Tannins-Rich Extract from Alder Bark for Particleboard and Plywood Production

**DOI:** 10.3390/ma15113894

**Published:** 2022-05-30

**Authors:** Sarmite Janceva, Anna Andersone, Uldis Spulle, Ramunas Tupciauskas, Electra Papadopoulou, Oskars Bikovens, Martins Andzs, Natalija Zaharova, Gints Rieksts, Galina Telysheva

**Affiliations:** 1 Latvian State Institute of Wood Chemistry, Dzerbenes Street 27, LV-1006 Riga, Latvia; jancevasarmite@gmail.com (S.J.); ramunas.tupciauskas@kki.lv (R.T.); oskars.bikovens@kki.lv (O.B.); martins.andzs@kki.lv (M.A.); natalija.zaharova@gmail.com (N.Z.); gints.rieksts@inbox.com (G.R.); telysheva@gmail.com (G.T.); 2 Ekokompozit Ltd., Dzerbenes Street 27, LV-1006 Riga, Latvia; 3 Department of Wood Processing, Latvia University of Life Sciences and Technologies, Liela Street 2, LV-3001 Jelgava, Latvia; uldis.spulle@llu.lv; 4 Chimar Hellas S.A., 15 km National Road Thessaloniki—Polygyros, 570 01 Thessaloniki, Greece; papadopoulou@ari.gr; 5 The Institute of Physics, University of Latvia, Miera Street 32, LV-2169 Salaspils, Latvia

**Keywords:** alder bark, extract, condensed tannins, phenol-formaldehyde resin, formaldehyde-free, green adhesive, formaldehyde emission, plywood, particleboard, composite materials

## Abstract

Toxic formaldehyde emissions, and the necessity to reduce the consumption of petrochemicals, stimulates the development of environmentally friendly adhesives. The aim of this research was to study, for the first time, the possibility of using condensed tannins (CTs)-rich extracts from grey alder (*Alnus incana*) and black alder (*Alnus glutinosa*) bark in the production of particleboards and plywood adhesives. The chemical structure, composition, and molecular weight of the CTs were identified by a ^13^C-NMR and TOF-MS analysis. Three innovative adhesive systems were studied: CTs-phenol-formaldehyde (CTs-PF) resin; a CTs-polyethyleneimine (PEI) adhesive system; and CTs–PEI combined with an ultra-low emitting formaldehyde resin (ULEFR)—CTs–PEI–ULEFR. The results showed that CTs-PF resin has properties close to commercial PF resin, and the formaldehyde emission was twice lower. CTs–PEI bonded particleboards corresponded to the requirements of the EN 312:2010 standard for particleboards in dry conditions (Type P2). CTs–PEI–ULEFR, with a 40–60% substitution of ULEFR by CTs–PEI, had adhesive properties very close to ULEFR; the plywood shear strength fit the requirements of the EN 314-2:1993 standard for application in internal and external system conditions. The introduction of extracted alder bark residues microparticles into the composition of the adhesive system showed their positive potential for application as a filler.

## 1. Introduction

Phenol-formaldehyde resins (PFRs), which are products of polymerization of phenolic compounds with formaldehyde, have a significant share in the total global production of synthetic resins. The PFRs are low cost, have available sources of raw materials, are easy to obtain and modify to control certain properties, and have a wide range of operational purposes. The world production of PFRs in 2020 amounted to about 12.3 million tons; the global phenolic resins market was valued at $8.2 billion in 2020 and is projected to reach $12.6 billion by 2030 [1].

In Europe, the largest producers of PFRs (urea-, phenol- and melamine-formaldehyde), which are used in the production of plywood, fiberboard, chipboard, oriented standard board, and other wood-based materials, are Germany, Finland, and Norway. The PFRs-based wood composite materials have good mechanical properties, weather and chemical resistance, and thermal stability [2,3]. The disadvantages of PFRs are the toxicity of their main components at production and emissions from the PFRs-containing composite materials [4]. Numerous investigators reported the hazardous effect of formaldehyde on human health; children, allergy sufferers, and the elderly are the most sensitive. A high concentration of formaldehyde in the air can produce spasms, edema of the larynx, and increase the risk of cancer [5,6]. Furthermore, unreacted formaldehyde can remain in the PF resin products, leading to its potential low level, but long-term exposure in homes, kindergartens, hospitals, nursing homes, offices, and other places where PF products are commonplace. There are different formaldehyde emission standards all over the world and the regulations are difficult to compare as they are based on different testing methods [7]. However, the common tendency for all of them is to gradually lower the emission limit since, in 2006, the International Agency for Research on Cancer (IARC) reclassified formaldehyde from “probable human carcinogen” (Group 2A) to “carcinogenic to humans” (Group 1). All standard regulations are based on the analysis of the wood composites and not the adhesive used. Since 2004, based on harmonized European Standard (EN 13986 2015), emission classes E1 (≤8 mg/100 g) and E2 (8–30 mg/100 g) are used. In 2016, the European Low Emission Standard (E.LES) set different emission limits for different product types; it set a lower value for particleboard and plywood (0.065 ppm). In Germany, the European E1 emission level of 0.10 ppm was decreased to 0.05 ppm, which is considered a limit for ULEF emission. In Germany, Austria, Denmark, and Sweden, formaldehyde concentration limits of 6.5 mg/100 g dry board are obligatory. Therefore, the maximum replacement of formaldehyde in the adhesive system, or its full replacement by another type of adhesive is a necessary task [7]. In addition, the source of PF production is petroleum, but the replacement of non-renewable fossil resources, as well as an increasing independence from them, is a necessary goal. Apart from PF, urea-formaldehyde (UF) and melamine-urea-formaldehyde (MUF) resins, other adhesive systems such as polyurethanes (PUR) are gaining in popularity. PUR adhesives exhibit excellent joint strength in standard climate conditions, but seem not to be so reliable in the case of low wood moisture content or under high temperatures [8]. Polyvinyl acetate (PVAc) glue is another alternative bonding agent. Unfortunately, its high viscosity makes it difficult to apply using methods for urea-formaldehyde glues. The study showed that the bending strength and internal bond strength of particleboards produced with the use of PVAc glue are lower than for panels produced with the use of UF resin [9]. In recent years, a considerable amount of research has been done on natural adhesives from sustainable resources such as lignin [10,11,12], soybean [12,13,14], condensed tannins [15,16,17,18], and suberinic acids [19] in the partial of full replacement of PF. Condensed tannins (CTs) from lignocellulosic biomass could potentially be eco-friendly substitutes for phenol in adhesives due to the high reactivity of CTs toward aldehydes, including formaldehyde [20]. In a recent study, CTs have been suggested as scavengers for formaldehyde in melamine-formaldehyde (MF) resin [15]. The application of ground birch (*Betula verrucosa Ehrh.*) bark as an eco-friendly additive in urea-formaldehyde (UF) adhesives for plywood manufacturing showed successful results as a formaldehyde scavenger in plywood production, mainly due to its tannin content [21].

**Figure 1:**
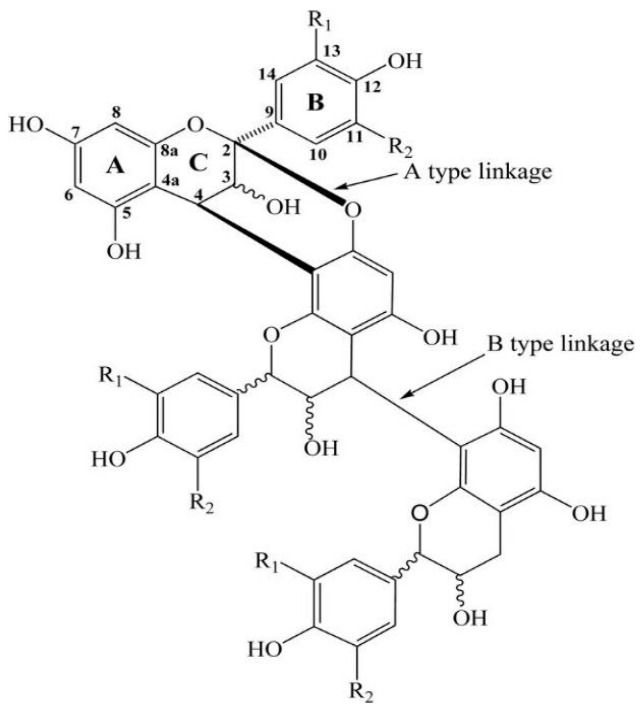
A- and B-type oligomeric chemical structure of CTs (R1 = OH, R2 = H, procyanidin) [28].

CTs are present in large concentrations in softwood and hardwood bark [22,23]. CTs-based resin research and its application have been successful in some countries, e.g., Australia, Chile, Argentina, Brazil, and New Zealand [24]. Quebracho and acacia tree extracts constitute the main part of the bulk CTs production for leather tanning [25]. Their extracts are composed of approximately 70% CTs and 20–25% non-tannins compounds, mainly simple sugars that contribute negatively to the final properties of PFR. Some commercialization trials of CTs-based adhesives indicated problems such as high viscosity at the concentrations normally required in adhesives and low moisture resistance [26,27]. CTs are chemically heterogeneous oligomers and polymers of flavan-3-ol monomer units linked mainly by C4–C6 or C4–C8 bonds (B-type of CTs). Less widespread are the A-type CTs, characterized by the presence of flavanol units double-linked by C4–C8 and C2–O7 or C4–C6 and C2–O7 bonds (Figure 1) [28].

It has been established earlier [29] that the bark of black alder (*Alnus glutinosa*) and grey alder (*Alnus incana*), the tree species widely spread in European countries including the Baltic region, contain CTs in rather large quantities (about 10–12% on oven-dry bark). The study of the alder bark application in adhesives has already been undertaken by the authors, and the preliminary results showed the potential of grey alder, black alder, ash tree, and goat willow CTs in gluing particleboards and plywood, which raised the question of more profound research and testing according to those standards. The high reactivity of CTs with other formaldehyde-free polymers, such as rubber [24,30], polyethyleneimine (PEI) [31], polymer diphenylmethane diisocyanate (pMDI) [17], and PVAc [32], have shown its high contribution and suitability for obtaining new eco-friendly wood adhesives. The present study continues our work with alder bark CTs [33,34] by illustrating the potential of grey alder, black alder, ash tree, and goat willow CTs for gluing particleboards and plywood, and is focused on the innovative research of a hydrophilic alder bark extracts containing oligomeric CTs as the main component of adhesives in three ways: (1) partial phenol substitution in the synthesis of PFR; (2) the modification of CTs with PEI, as a no-added-formaldehyde (NAF) adhesive system; (3) and the combination of CTs–PEI-ultra-low emitting formaldehyde. For the creation of the potential waste-free production process, the alder bark residues after the extraction of CTs were tested as a microparticle filler in the composition of the adhesives, for the improvement of physical–mechanical properties and bonding strength.

## 2. Materials and Methods

### 2.1. Materials

#### 2.1.1. Alder Bark

The bark (inner and outer bark mix) from the grey alder (*Alnus incana* (L.) Moench, further in the text—*Alnus incana*) and black alder (*Alnus glutinosa* (L.) Gaertn, further in the text—*Alnus glutinosa*) was collected in a forest in the south-eastern part of Latvia. The age of cut alder trees was between 25 and 30 years. The debarking of the alder tree (*Alnus incana* and *Alnus glutinosa*) logs was carried out with 4 m^3^ of logs with a diameter of 26–42 cm and a length of 3.1 m (approximately 10 trees). Trees were cut from an area of 17 ha.

The bark was dried at room temperature, ground with a knife mill (Retsch SM100, RETSCH, Haan, Germany), and sieved to select the particles between 2 and 4 mm.

#### 2.1.2. Chemicals

The analytic standard procyanidin B2 (purity ≥ 90% (HPLC), and reagents: FeNH_4_(SO_4_)_2_∙12 H_2_O, n-butanol (purity ≥ 99.4%), and Sephadex LH-20 were purchased from Aldrich Sigma. The 50% (*w*/*v*) polyethyleneimine–water solution (*M*w = 1300) was purchased from Sigma-Aldrich (Sigma-Aldrich Chemie GmbH, Steinheim, Germany) and used as received.

#### 2.1.3. Pine Particles for Particleboards

Pinewood particles with a particle size of 1–2 mm were contributed from JSL Serviss Ltd. (Riga, Latvia) Freshly collected pinewood sawdust was air-dried at room temperature for one week to 10% of moisture, and 1 day before contact with the adhesive they were additionally dried in the drying oven until they reached 2–4% a moisture level and then were put into a hermetically sealed bag.

#### 2.1.4. Veneer Sheets for Plywood

Veneer sheets of rotary birch wood *(Betula pendula)* were contributed by the Forest and Wood Products Research and Development Institute. The veneer was conditioned at 20 °C and 65% relative humidity for one week. The thickness of the birch peeled veneer sheets was 1.5 ± 0.2 mm. The ULEFR was contributed by Latvijas Finieris AS (Riga, Latvia).

### 2.2. CTs-Rich Extract Isolation from Grey and Black Alder Bark

The CTs-rich extracts were obtained from the alder bark by reflux extraction with 50% (*v*/*v*) EtOH, and by water extraction, at 80 °C for 60 min. The extracts were freeze-dried using lyophilization equipment at Heto Power Dry HS3000 (Thermo Fisher Scientific, Waltham, MA, USA) to yield a brown solid. The CTs-rich extract was stored at −8 °C (experimentally proved chemical stability during at least a year).

### 2.3. Determination of CTs Content in the Extract

CTs content in the extracts was measured by the butanol–HCl method [35] using procyanidin dimer B2 as a reference compound. Amounts of 6 mL of acid butanol (5% (*v*/*v*) concentrated HCl in n-butanol) and 0.2 mL of iron reagent (*w*/*v*) (FeNH_4_(SO_4_)_2_∙12 H_2_O in 2 M HCl) were added to 1 mL of the extract aliquots whilst stirring the tube without heating and allowing it to be heated in a water bath at 80 °C for 50 min. After 50 min, the absorbance of the mixture was measured against a blank solution at 550 nm using a UV/VIS spectrometer Lambda 650 (Perkin Elmer, Inc., Waltham, MA, USA).

Each extract was analyzed in triplicate, and assay results were expressed as a percentage per oven-dry (o.d.) extract. The confidence interval (CI) for the results did not exceed 3% at α = 0.05.

### 2.4. Purification of CT_S_ from Non-Tannin and Sugar

The purification of CTs from non-tannin and sugar was carried out using a Sephadex LH-20 column with 96% EtOH and 70% (*v*/*v*) acetone as the respective purification solvents. In the purification process, low-molecular-weight phenolics were eluted with 96% EtOH, and the CTs were eluted with 70% (*v*/*v*) acetone. Purified CTs were evaporated using a rotary evaporator (Heidolph Instruments, Schwabach, Germany) prior to being freeze-dried and stored at −8 °C.

### 2.5. Determination of Carbohydrate Content in the Extract

The total amounts of the carbohydrate in the extracts were determined using GC analysis after hydrolysis, reduction, and acetylation as described by Janceva et al. [35]. Gas chromatographic analysis was performed using an Agilent 6850 Series: column—DB-1701; length—30 m; internal diameter—0.25 mm; layer thickness—0.25 µm. Each extract was analyzed in triplicate, and assay results were expressed as a percentage per o.d. extract. The confidence interval (CI) of the results did not exceed 3% at α = 0.05.

### 2.6. Characterization of Purified CTs

The ^13^C-NMR spectra of the purified CTs were recorded with a Bruker Avance 300 spectrometer (Bruker Corporation, Billerica, MA, USA). The CTs sample was dissolved in D_2_O (ca. 2% *w*/*w*). The TOF-MS spectra of CTs were recorded with a Qstar Elite System Hybrid Quadrupole-TOF/MS spectrometer (Qstar Elite, Applied Biosystems, Waltham, MA, USA).

### 2.7. Preparation and Characterization of CTs-Based Adhesive Systems

#### 2.7.1. CTs–PEI Adhesive

The CTs–based no-added-formaldehyde (NAF) adhesive system was obtained by mixing of aqueous 20% CTs rich extract solution (pH 7) with 50% (*w*/*v*) aqueous polyethyleneimine (PEI) solution (optimal mass ratio 2:1, *w*/*w* [31]). The resulting adhesives were used to produce particleboards and plywood.

#### 2.7.2. CTs–PEI-ULEFR Adhesive 

Adhesives for plywood manufacture were made by mixing CTs–PEI (pH 10) mixture with ULEF resin at the mass ratios (CTs–PEI):ULEFR = 2:8, 4:6, 5:5, 6:4, 8:2. The resulting adhesives were used to produce plywood.

#### 2.7.3. Synthesis of a PF Resin with Phenol Substitution by CTs-Rich Extract

For the synthesis of the phenolic resins, phenol 90%, and formaldehyde water solution 37.4% were used. A PFT resin with 20% phenol replacement by CTs-rich extract was produced smoothly following the CHIMAR proprietary process (CHIMAR Hellas S.A., Thessaloniki, Greece) [36,37] for the synthesis of a PF-type resin.

### 2.8. Determination of pH

The pH of the obtained adhesives was measured using an automatic titration device Radiometer analytical CDM 210 (Hach Company, Loveland, CO, USA), which includes a 20 mL burette, a pH glass electrode, and a reference electrode Ag/AgCl. A three-point calibration was performed with a measurement range of 0–14 pH units with a resolution of ±0.002 pH units.

### 2.9. Determination of Adhesive System Viscosity and Gel Time 

The HAAKE 6 plus rotational viscometer (Thermo Scientific, Waltham, MA, USA) was used to determine the viscosity of the adhesive. ~50 mL of sample was poured into a thermostatic vessel and the rotating disk of the apparatus was immersed in it. The viscosity was determined at 25 °C with a range of 1–3,000,000 mPa∙s and spindles L1, L4.

The gel time of the CTs–PEI adhesives was determined on a tile at a temperature of 100 ± 3 °C.

### 2.10. Determination of Dry Matter Content of Adhesive

The dry matter content of the adhesives was determined by drying the samples in an oven at 105 ± 3 °C to constant weight.

### 2.11. Thermogravimetric Analysis

A thermogravimetric analysis for adhesive samples was performed using an STA 6000 Perkin Elmer (Perkin Elmer, Inc., Waltham, MA, USA) in an inert atmosphere at a temperature range of 20–650 °C and a heating rate of 10 °C. The initial weight of the sample was 15.20 mg. The CTs and PEI were analyzed separately to determine the temperature at which the thermal destruction of raw materials occurred.

### 2.12. Manufacture and Testing of Wood Composites

#### 2.12.1. Particleboard Production

The particleboards were made of pine wood chips (moisture content = 3%) and CTs–PEI adhesive (10% and 20% adhesive of the total dry weight). A rotary mixer was used to ensure the uniform blending of the adhesive and wood chips, and the adhesive system was added to the chips gradually for 5 min. The hot-pressing of the blended furnish was performed in a hot press “Joos” (Joos LAP 40, Gottfried Joos Maschinenfabrik GmbH & Co. Pfalzgrafenweiler, Germany) at 150 °C, according to the developed mode:

- Mat formation: pressure 1.3 MPa, time 2–3 min;
- Cycle 1—pressure 3 MPa, time 5 min;
- Cycle 2—pressure 2 MPa, time 4 min;
- Cycle 3—pressure 1.5 MPa, time 5 min;
- Cycle 4—vapor pressure reduction at 0.2 MPa in 30 s.

The particleboard dimensions were 350 × 350 × 10 mm. At the end of the pressing cycle before the press opening, the pressure was reduced to zero to avoid the formation of steam pressure within the pressed board above atmospheric pressure, which could cause cracks during the press opening [38]. The obtained particleboards were conditioned for 24 h at a temperature of 20 ± 2 °C and a relative humidity of 65 ± 5% before the testing of the physical and mechanical properties.

#### 2.12.2. Preparation of Plywood

Birch peeled veneer sheets with a size of 250 mm × 250 mm and a thickness of 1.5 ± 0.2 mm were used to obtain the plywood. The average density of the peeled veneers was 650 kg/m^3^. The plywood was formed from three veneer layers perpendicular to each other, consuming about 95 g/m^2^ of CTs–PEI adhesive and 170 g/m^2^ of CTs–PEI-ULEF adhesive.

The adhesive was applied to the birch veneer sheets with a roller. The formed veneer sheets were hot-pressed (JOOS LAP 150, Gottfried Joos Maschinenfabrik GmbH & Co., Pfalzgrafenweiler, Germany) in the following mode:

- pressing temperature 140 °C;
- pressing pressure 2 MPa;
- holding time under pressure—10 min.

The thickness of the produced 3-ply plywood was 4.0 ± 0.3 mm. After their manufacturing, the plywood samples were conditioned for 24 h at a temperature of 20 ± 2 °C and relative humidity of 65 ± 5%. 

#### 2.12.3. Preparation and Determination of the Adhesive Bonding Quality

**Figure 2:**
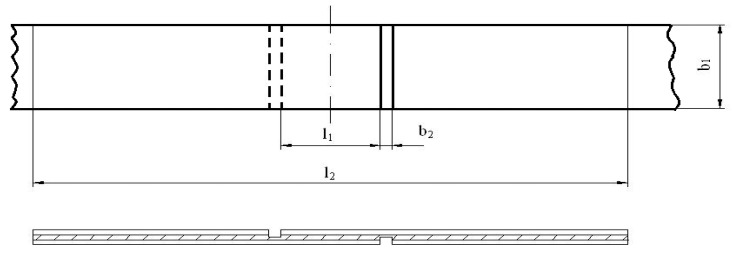
Shape and dimensions of strength test sample for 3 ply veneer plywood (l_1_—shear length (25 ± 0.5 mm); b_1_—shear width (25 ± 0.5 mm); b_2_—saw cut width (2.5 to 4 mm); l_2_—minimum distance between the clamps (50 mm).

After conditioning, the plywood samples were prepared for a determination of their bonding quality according to the EN 314-1:2014 standard [39], with a total specimen number of 10 for each adhesive system. The shape and dimensions of the specimens were prepared for testing each glue line between veneer sheets according to the requirements of the EN 326-1:1994 standard [40], as shown in Figure 2.

Before the bonding quality test, the plywood specimens were pre-treated in water according to the standard EN 314-2:1993 standard [41] to determine bonding classes, which suggest the following plywood application conditions: 

Class 1—dry conditions. Adhesives in this class are suitable for normal room conditions; 

Class 2—wet conditions. Adhesives in this class of adhesives are intended for external conditions in the absence of direct weathering (snow, rain), such as behind the external cladding or under the roof. Adhesives in this class are also resistant to short-term weather conditions; 

Class 3—external conditions. Adhesives in this class are suitable for outdoor use under prolonged exposure to external weather conditions.

For the all-bonding classes, each glue line was tested to meet 2 criteria as shown in Table 1.

CTs–PEI- and CTs–PEI-ULEFR-bonded plywood samples have been tested only for bonding classes 1 and 2, while CTs-PF-bonded plywood samples, obtained from CHIMAR laboratory, were tested for all bonding classes.

Before the specimen’s pre-treatment, the length and width of the shear area were measured using a digital caliper and label. During the pre-treatment of the specimens for bonding class 1, they were soaked in water (20 ± 3 °C) for 24 h. To determine the quality of the adhesive for bonding class 2, the specimens first were immersed in boiling water for 6 h and then cooled in water at a temperature of 20 ± 3 °C for at least 1 h. To determine the quality of the adhesive for bonding class 3, the samples were immersed in boiling water for 4 h, followed by drying in an oven at 60 °C for 16 to 20 h, followed by repeated boiling in water for 4 h, and then cooling in water for at least 1 h at a temperature of 20 ± 3 °C.

The shear strength test was performed for wet samples after the excess water was drained. The specimens were clamped in the jaws of an auxiliary test machine so that the force was applied centrally without the application of a transverse force in the shear plane. The force was applied uniformly at a constant speed so that the rupture occurred within 30 ± 10 s. The shear strength *f_v_* (N/mm^2^) of each specimen sample was determined in accordance with standard EN 314-1:2014 requirements. 

#### 2.12.4. Bending Test

The modulus of elasticity (MOE) and modulus of rupture (MOR) were determined for particleboard and plywood samples in a three point static bending test according to the EN 310:1993 standard [38] using universal testing equipment (ZWICK Z100, ZwickRoell Testing Systems GmbH, Fürstenfeld, Austria).

The internal bond or tensile strength perpendicular to the plane of the particleboard was determined by standard EN 319:1993 requirements [42]. After the additional steel blocks were glued to the sample surfaces by an adhesive, the test samples at a temperature of 20 ± 2 °C and relative humidity of 65 ± 5% were conditioned until they reached a constant mass.

### 2.13. Determination of Density

The density of wood composites was determined based on the standard requirements of EN 323:1993 [43].

### 2.14. Determination of Formaldehyde Emission from Plywood Samples

Formaldehyde emissions from CTs-PF plywood samples were determined based on the requirements of Japanese Industrial Standard JIS A 1460:2001 [44].

**Figure 3:**
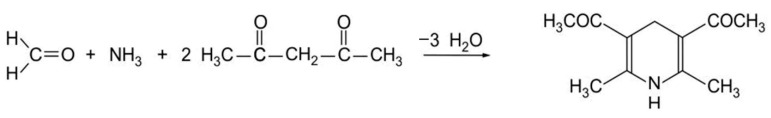
Reaction for formaldehyde determination.

The plywood samples were placed in a glass desiccator for 24 h at 20 °C. During this time, formaldehyde accumulated in the water. The determination of the formaldehyde concentration is based on reaction, where formaldehyde reacts with ammonium ions and acetylacetone to produce 3,5-diacetyl-1,4-dihydrolutidine (DDL) (Figure 3). Formaldehyde emissions were expressed in mg/L.

Formaldehyde emissions from CTs–PEI-ULEFR plywood samples were determined based on the requirements of standard EN 717-2: 1994 [45]. It is a gas analysis method in which formaldehyde emissions are measured from the surface of the plywood, where the edges of the plywood were sealed with self-adhesive aluminum tape.

### 2.15. Preparation and Determination of Particle Size of Extracted Alder Bark Residue

To evaluate the effect of the extracted alder bark as a filler on the mechanical properties of CTs–PEI-ULEF adhesive for plywood production, the alder bark after water extraction was crushed using a Roller ball mill TMAX-PM-2 (TMAX, Xiamen, Fujian, Mainland, China). The particle size of the crushed bark was determined using a Zetasizer Nano ZS 90 (Malvern Panalytical Ltd., Malvern, UK).

### 2.16. Statistical Analysis

The chemical characterization of the CTs and CTs-based adhesives were conducted in triplicate, and the results are presented as the mean value ± standard deviation (SD). Statistical analyses were performed using Microsoft Excel 2016. Confidence intervals (CI) for a mean using a Student’s T-distribution were calculated at a significance level of α = 0.05.

10 samples were taken for each particleboard and plywood samples analysis. The lower 5% quantile was used to assess the non-uniformity of the plywood and particleboard results. The lower 5% quantile was calculated according to Equation (1):

(1) $$L_{5\%}^{q}=x-t\cdot s,$$

where: *x*—average value of measurements; *t*—coefficient, which depends on the number of samples (10 samples −1.89); *s*—standard deviation (SD).

## 3. Results and Discussion

### 3.1. Chemical Characterization of Hydrophilic Extracts and CTs from Alnus Incana and Alnus Glutinosa

**Figure 4:**
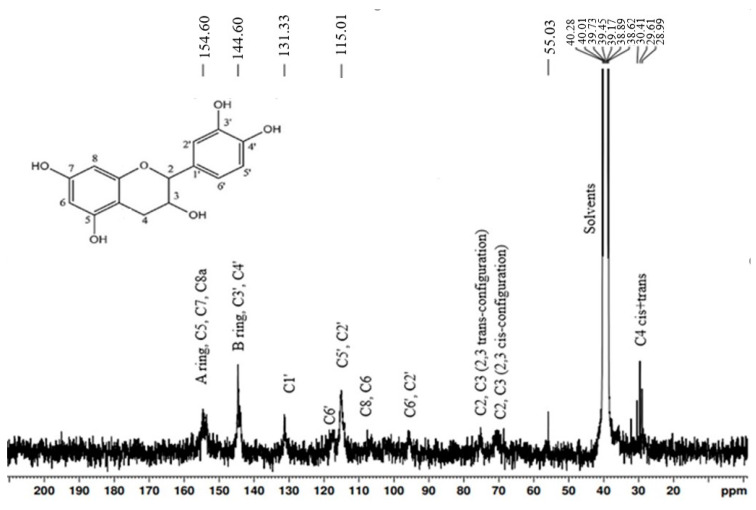
The ^13^C-NMR spectrum of purified CTs from *Alnus incana* bark.

**Figure 5:**
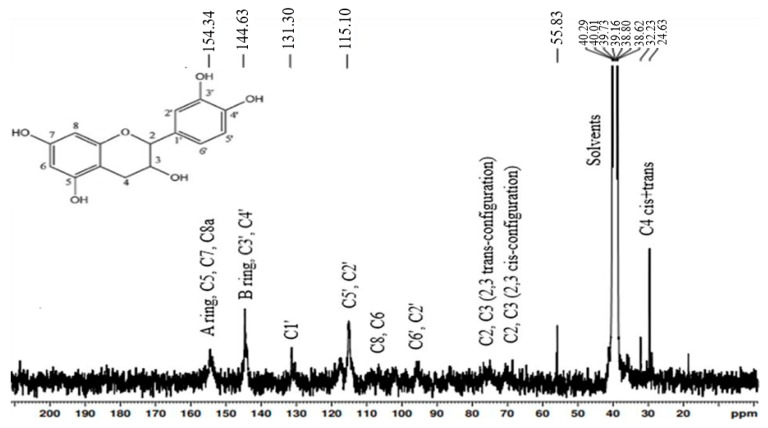
The ^13^C-NMR spectrum of purified CTs from *Alnus glutinosa* bark.

It was found that CTs are the major polyphenolic compounds of the 50% EtOH hydrophilic extract isolated from *Alnus incana* and *Alnus glutinosa* bark. The content of CTs in *Alnus incana* and *Alnus glutinosa* 50% (*v*/*v*) EtOH extracts was 42 and 46% of the o.d. extract, correspondingly, and the content of CTs in the water extract of *Alnus glutinosa* was 24% of o.d. extract. The gas chromatography results showed that the hydrophilic extracts contain a certain amount of carbohydrates—24% for *Alnus incana* 50% EtOH extract, 26% for *Alnus glutinosa* 50% EtOH extract, and 33% for *Alnus glutinosa* water extract. The CTs-rich extracts were purified from the non-tannin compounds, and for the chemical characterization of the CTs’s composition, structure, and molecular weight. The purification of CTs was carried out using sorbent Sephadex LH-20. The freeze-dried CTs were analyzed by ^13^C-NMR and TOF-MS. ^13^C-NMR spectra (Figure 4 and Figure 5) of the purified CTs provided detailed information on the substructures and functional groups of the tested compounds, which allowed to identify the chemical structure of the analyzed CTs.

The interpretation of the ^13^C-NMR spectral signals was performed based on literature data [46], indicating that the analyte consists of CTs catechin or epicatechin units connected by C4–C8 and C4–C6 inter-flavonoid bonds that are characteristic for B-type CTs, and double-bond flavanol units in the molecule (C2-O-C7), which is typical for A-type CTs. The characteristic ^13^C signals of CTs were C5, C7, and C8a carbons between 160 and 150 ppm. Signals at 142–145 ppm belong to C3 and C4, and peaks at 129–132 ppm to C1. The presence of signals at 30 ppm can be assigned to C4. The cluster of signals between 110 and 95 ppm can be assigned to C8, C6, C6′, and C2′. The signal at 50–60 ppm showed the presence of carbohydrates in purified CTs. According to the gas chromatography results, the content of remaining carbohydrates in the form of glycosides was 4%.

**Figure 6:**
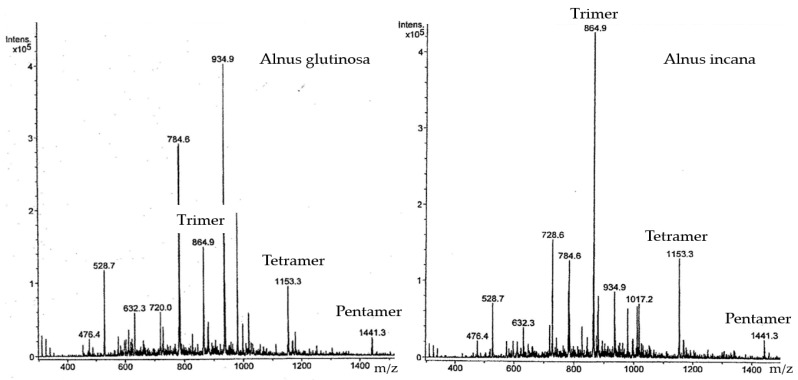
TOF-MS spectra of CTs from *Alnus glutinosa* and *Alnus incana* bark.

Based on the TOF-MS mass spectra (Figure 6), the molecular weight of CTs in *Alnus incana* and *Alnus glutinosa* extract did not exceed 1441 Da. Regular fragmentation by [M-H]^−^ *m*/*z* = 288 corresponding to the catechin/epicatechin units indicates that CTs contain catechin/epicatechin trimers ([M-H]^−^ *m*/*z* = 865), tetramers ([M-H]^−^ *m*/*z* = 1153), and pentamers ([M-H]^−^ *m*/*z* = 1441).

### 3.2. Evaluation of Plywood Produced with CTs-PF Resin Subsection

For the testing of the CTs-rich extract as a phenol substitute in the standard PFR system for plywood production, 20% of phenol was replaced by the CTs-rich extract from *Alnus incana* following the CHIMAR Hellas S.A methodology [37,39]. The results (Table 2) showed that the obtained CTs-PF resin has properties close to the standard PF resin and passes the threshold values according to EN 314-2:1993 standard [41]. The plywood made with the CTs-PF resin showed almost twice the lower formaldehyde emission value than the plywood produced with a standard PF resin.

### 3.3. Characterization of CTs-Based NAF Adhesive System

The CTs-rich extracts from *Alnus incana* and *Alnus glutinosa* were modified with a 50% PEI aqueous solution to obtain new, environmentally friendly formaldehyde-free adhesives for wood materials. 

**Figure 7:**
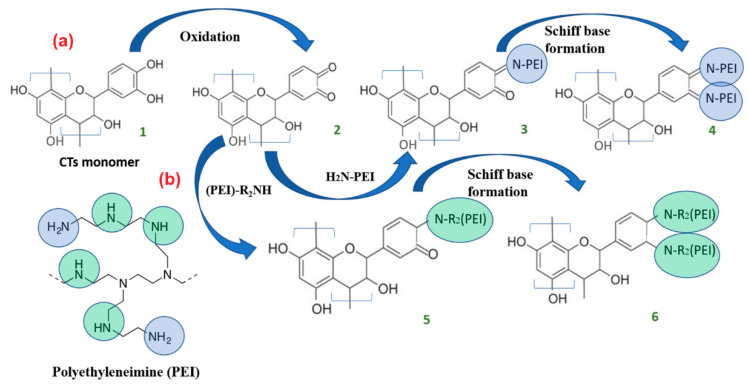
The possible schemes of the reaction between CTs and primary (**a**) and secondary amine (**b**) groups of PEI.

It is known [47] that the catechol moiety, namely, the B-ring structure of CTs, is highly sensitive to oxidation, resulting in the formation of ortho-quinone. The C = O groups in ortho-quinone readily react with terminal primary amine groups (–NH_2_) of PEI to form Schiff bases 3 and 4 (Figure 7) and with secondary amine groups (R_2_NH) dominantly presented in PEI forming enamines [48].

**Figure 8:**
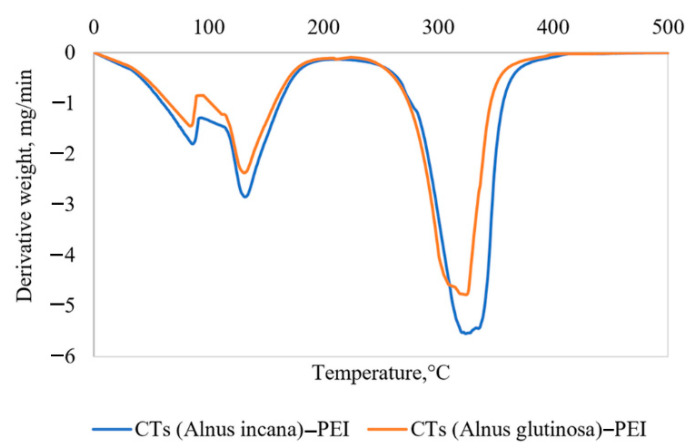
Differential thermogravimetric curves of *Alnus incana* and *Alnus glutinosa* bark CTs–PEI adhesive system.

The thermal stability of the CTs–PEI adhesives was studied by the TGA method. The CTs–PEI adhesives degradation revealed three main regions of mass losses (Figure 8).

The first region of the spectra with the peak degradation rate achieved at 85 °C is associated with the loss of water as a solvent, and the second region with the peak rate at 132–133 °C (for *Alnus incana* and for *Alnus glutinosa,* correspondingly) is attributed to the release of volatile compounds such as chemically bonded water, CO, and CO_2_ formed as the result of thermally unstable components (predominantly carbohydrates) degradations [49], as well as partial degradation of low-molecular PEI fractions. The complex chemical structure of CTs and PEI adhesive demonstrates the high heat thermal resistance. This is confirmed by the third peak of degradation rate at 324 °C (for *Alnus incana)* and 322 °C (for *Alnus glutinosa)* where the major mass loss of the adhesive (scission of chemical bonds in products of condensation) was reached. The results obtained by the thermogravimetric analysis confirmed that the selected hot-pressing temperature (140–150 °C) for particleboard and plywood production was acceptable.

**Figure 9:**
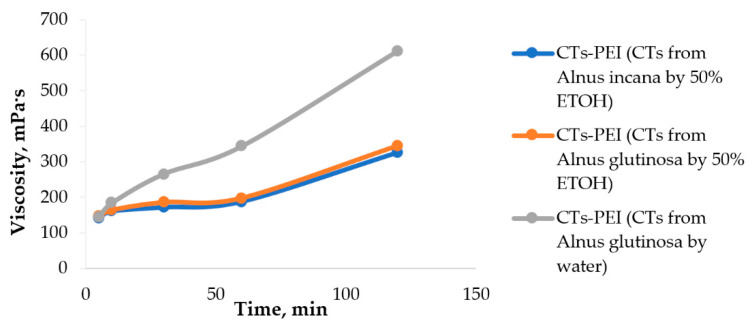
The viscosity of CTs–PEI adhesive depending on time, at 25 °C.

The experimentally obtained CTs–PEI adhesives were a viscous liquid, brown in color, with a dry matter content of 30% without a specific odor. The viscosity of the CTs–PEI adhesives increased with time (Figure 9).

Adhesive bonding performance between wood elements, for example, veneer sheets, is presumed to be significantly influenced by the degree of penetration of the adhesive into the anatomic porous network of wood material cells. CTs–PEI adhesives with a viscosity of 172–198 mPa·s had a relatively low penetration into the wood, which increased the chemical bonding between the wood surface and the adhesive. The extract itself (20% CTs-rich extract solution) was also tested without the addition of PEI. Despite the stickiness of the extract, it was of too low viscosity, its main part almost completely penetrated into the irregularities and anatomical pores of the wood material cells, and the remaining amount was not enough to form a continuous sticky film. Increasing the amount of extract is impractical since hot pressing will consume too much energy to evaporate water. 

The pH of the CTs–PEI adhesive systems was about 10. Gel time, the second main characteristic of the adhesive, was defined as the point at which the product formed by the reaction of CTs and PEI becomes an elastic and rubbery solid. The gel time of the CTs–PEI adhesives was determined on a tile at a temperature of 100 ± 3 °C. When withheld at this temperature, the adhesives lost their plasticity, and for the ones obtained on the basis of CTs from alder bark extraction by 50% EtOH, after 10 min the gel point of the adhesives was reached. For the adhesive based on CTs obtained by water extraction, the gel point was reached in the first two minutes. Such a short temperature can create difficulties in the practical application of this adhesive. 

### 3.4. Particleboards Properties

The particleboards were made of pine wood particles by mixing them with CTs (20% aqueous solution) and CTs–PEI adhesive systems, with adhesive consumption of 10 to 20% of total dry pinewood particle mass. The particleboards were similar to commercial ones, with a density range of 1055 ± 45 kg/m^3^.

The obtained values of particleboard bending properties, depending on the used adhesive, are given in Table 3 and compared with the requirements of the standard EN 312:2010 for particleboards used in dry conditions (Type P2) [50]. 

For all adhesive systems under study, the particleboard bending values improved with increasing the applied adhesive content (10% or 20%). For the bending properties of the particleboards bonded with 10% adhesive content, no significant differences were observed for different CTs extraction methods and raw material. Increasing the adhesive content to 20% resulted in the best bending properties for the particleboards obtained with ethanol-extracted CTs from both *Alnus incana* and *Alnus glutinosa* mixed with PEI. These particleboards fully fit the strength modulus (MOR) standard requirement; however, the values of elasticity modulus (MOE) were too low to meet the standard due to the lower 5% quantile (Table 3). The best adhesive formulation used in this study for particleboard production was CTs–PEI based on 50% ethanol-extracted bark of *Alnus glutinosa,* with an adhesive system that amounted to 20% of pinewood particle mass. This was consistent to some extent with the results of other studies [34], supplementing them by revealing the innovative source for valuable CTs extraction (alder tree bark). For this specimen, the internal bond (IB) was determined, and it fits into standard EN 312:2010 P2 requirements.

**Figure 10:**
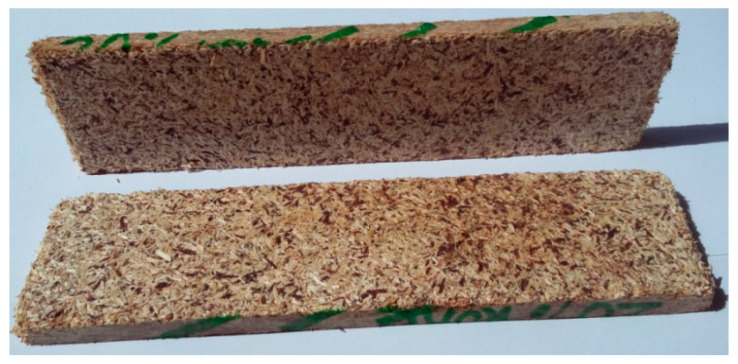
Particleboard specimens before bending test.

Adhesives on the basis of CTs obtained by 50%EtOH extraction (CTs content of 42–46% on o.d. extract), compared with the adhesives on the basis of CTs from water extraction (23% yield of CTs on o.d. extract), had higher adhesion properties, and thus the mechanical characteristics—MOR values of particleboards (Table 3). The thickness of the produced particleboards was 10 ± 0.2 mm (Figure 10).

### 3.5. Plywood Properties

The CTs–PEI adhesive systems were also tested for birch veneer bonding. The average consumption of CTs–PEI adhesive (with 30% of total solid content, viscosity 172–189 mPa·s) for plywood production was 95 g/m^2^. Birch peeled veneer sheets with a size of 250 mm × 250 mm and a thickness of 1.5 ± 0.2 mm were used to obtain the plywood. For testing of obtained plywood mechanical properties, the samples were pre-treated according to the requirements of standard EN 314-2:1993 [41]. The result of the gluing quality tests of the birch plywood panels glued by CTs–PEI adhesive showed that the mechanical properties (modulus of elasticity) of the glued veneer were close to the ones glued with PF resins (Table 3). However, wet shear strength was unsatisfactory (the panels disintegrated after an hour of pretreatment in water before the break, Class 1). For improvement of the properties, it is possible to make combinations of tested adhesives with more resistant and reactive adhesives or additives. Therefore, it was decided to combine CTs–PEI with ULEF resin, to improve plywood water resistance. Adhesive systems were prepared in different mass proportions of CTs–PEI: ULEFR (2:8; 4:6; 1:1; 6:4; 8:2). 

According to the literature, the polymerization between CTs (flavonoid unit) and formaldehyde mainly occurs on the A-ring through methylene (-CH_2_-) or methylene ether bridge linkages. Nucleophilic centers on the A-ring of any flavonoid unit tend to be more reactive than those on the B-ring [51]. 

**Figure 11:**
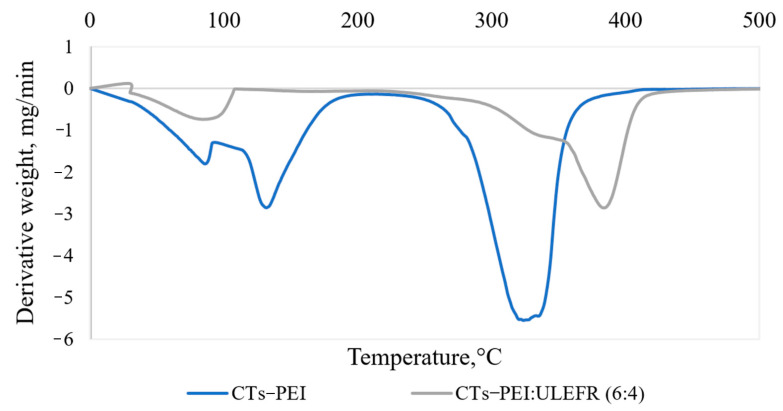
Differential thermogravimetric curve of the CTs–PEI-ULEFR and CTs–PEI adhesive systems.

The addition of ULEFR to the CTs–PEI adhesive improved the thermal stability of the adhesive system (Figure 11).

As in the above TGA spectrum, the first peak at the temperature of 87.4 °C indicates the evaporation of water. The thermal degradation of the CTs–PEI-ULEFR adhesive starts at about 220 °C, and the temperature of maximum degradation (maximum mass loss) rate was 320 °C for CTs–PEI.

The viscosity of the CTs–PEI adhesive was lower than that of ULEF resin. Replacing 20% of ULEFR by CTs–PEI causes a rapid increase in viscosity due to the high molecular weight of modified CTs and the existence of hydrogen bonding and electrostatic interactions between CTs and free formaldehyde molecules of ULEFR. By replacing 40% of ULEFR with CTs–PEI, the increase in viscosity was so big that it was not possible to measure the viscosity using a HAAKE Viscotester 6 plus viscometer. Such viscosity of adhesives is suitable only for plywood production. The viscosity and the results of the gluing quality tests of the birch veneer with all CTs–PEI-ULEFR adhesives prepared in different mass proportions of CTs–PEI: ULEFR, are shown in Table 4. The average consumption of CTs–PEI–ULEFR adhesive for plywood production was in the range of 170–230 g/m^2^.

**Figure 12:**
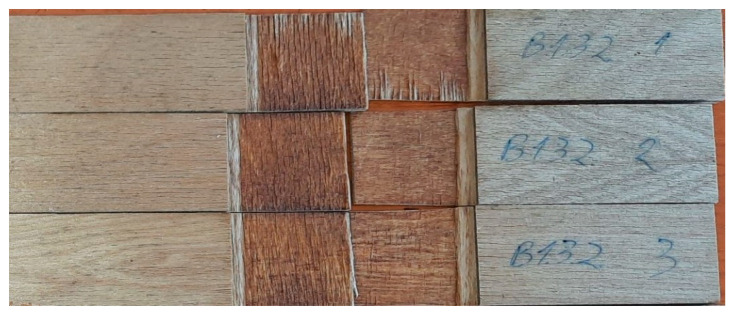
Scheme of the plywood specimen for the test (A) and experimentally obtained plywood specimen after the shear strength test.

The results showed that the values of modulus of elasticity (MOE) for plywood panels (obtained using the adhesive system with ULEFR substituted by 40–60% of CTs–PEI were close to those for the control panels glued by the ULEFR only. It was found that the values of share strength for these panels after treatment by immersion in water at 20 °C for 24 h (bonding class for dry interior) and cyclic treatment in boiling water (bonding classes for exterior application) pass the threshold values of ≥1 N/mm^2^ according to the requirements of standard EN 314-1:2014, clauses 5.1.3. and 5.1.1. After testing the plywood samples’ shear strength, the breaking force was determined according to the requirements of EN 314-1:2014, clause 5.2. For all plywood samples, the destruction took place over the adhesive line (Figure 12).

The emission of formaldehyde in plywood samples was determined in accordance with the requirements of the standard EN 717-2:1994. Formaldehyde emission for plywood CTs–PEI–ULEFR with mass proportions of CTs–PEI: ULEFR (6:2) was 0.027 mg/m^2^·h.

Introduction of alder bark extraction residues as microparticles (307 nm) filler into the composition of CTs–PEI–ULEF adhesive (10% on the adhesive mass), didn’t decrease the physical-mechanical properties and gluing strength for plywood (there was even a tendency to increase the value, but it was within the error, Table 5). Therefore, application of the remainders after extraction as a filler is possible and could be even desirable for diminishing the necessary amount of adhesive. Further experiments are necessary.

## 4. Conclusions

The oligomeric CTs are the dominant polyphenolic compounds in the composition of black alder (Alnus *glutinosa*) and grey alder (*Alnus incana*) 50% EtOH (*v*/*v*) and water extracts. Based on the chemical characterization of these CTs, it was found that they consist of catechin and epicatechin monomer units with a degree of polymerization of 3–5 related to bonds C4-C8 or C4-C6 (B-type of CTs) and C2-O-C7 (A-type of CTs).

Using these CTs-rich extracts from *Alnus incana* and *Alnus glutinosa* bark, three innovative adhesives for particleboard and plywood production were studied: CTs-phenol-formaldehyde (CTs-PF) resin; CTs-polyethyleneimine (CTs–PEI)—no-added-formaldehyde (NAF) adhesive system; combined CTs–PEI-ULEFR adhesive system. 

Plywood panels produced using CTs-PF resin (with 20% phenol substituted by CTs) may be used for interior and exterior construction system applications. Formaldehyde emissions from CTs-based plywood panels were twice lower than for PF-based plywood panels.

CTs–PEI adhesives most probably could be used for particleboard production for interior applications in dry conditions (Type P2) according to European standard EN 312 (2010). Further investigations are necessary to improve the modulus of elasticity, and the water resistance of such adhesives.

CTs–PEI–ULEFR adhesive system, with 40–60% substitution of ULEFR by CTs–PEI, had adhesive properties very close to typical PF resin; plywood shear strength fit the requirements of the standard EN 314-2:1993, for application in internal and external conditions. Due to the very low level of formaldehyde emission, the obtained CTs–PEI-ULEFR could be used for the production of plywood for interior and exterior applications. 

The introduction of extracted alder bark residues microparticles into the composition of the adhesives showed their potential for application as a filler in the adhesive system.

## Figures and Tables

**Table 1:** Plywood glue line bonding requirements.

Shear Strength, f_m_ (N/mm^2^)	Wood Failure, W (%)
0.2 ≤ *f_v_* < 0.4	≥80
0.4 ≤ *f_v_* < 0.6	≥60
0.6 ≤ *f_v_* < 1.0	≥40
1.0 ≤ *f_v_*	No requirements set

**Table 2:** Shear strength, wood failure, and formaldehyde emission of the plywood produced with CTs (20%)-PF resin, in comparison to plywood, produced with a standard PF resin.

Characteristics	Standard PF Resin	$L_{5\%}^{q}$	CTs (20%)-PF Resin	$L_{5\%}^{q}$
	**No treatment**			
**Shear strength, N/mm^2^**	2.58 ± 0.23	2.14	2.06 ± 0.31	1.47
**Wood failure, %**	60 ± 2	-	64 ± 1	-
	**Pre-treatment required by Class 1: immersion in water of 20 °C for 24 h**			
**Shear strength, N/mm^2^**	1.45 ± 0.22	1.03	1.62 ± 0.32	1.02
**Wood failure, %**	88 ± 2	-	60 ± 1	-
**Pre-treatment required by Class 3: 4 h in boiling water–16 h drying at 60 °C–4 h in boiling water–1 h in cool water**				
**Shear strength, N/mm^2^**	1.31 ± 0.16	1.01	1.14 ± 0.11	0.93
**Wood failure, %**	83 ± 2	-	37 ± 1	-
**Formaldehyde emission (based on the requirements of JIS A 1460:2001, mg/L**				
	0.090 ± 0.008	-	0.047 ± 0.009	-

**Table 3:** The bending properties of particleboard depending on the used adhesive system.

Adhesive System	MOR, N/mm^2^	$L_{5\%}^{q}$	MOE, N/mm^2^	$L_{5\%}^{q}$	IB, N/mm^2^	$L_{5\%}^{q}$
**CTs-rich extract from *Alnus incana* bark extracted by 50% EtOH (42% CTs on o.d. extract)**						
**CTs 20% aqueous solution (10% of pinewood particles mass)**	5.3 ± 1.4	2.7	819 ± 180	479	-	-
**CTs 20% aqueous solution (20% of pinewood particles mass)**	13.2 ± 3.2	7.2	1305 ± 293	751	-	-
**CTs–PEI (10% of pinewood particles mass)**	5.0 ± 1.1	2.9	699 ± 232	261	-	-
**CTs–PEI (20% of pinewood particles mass)**	16.9 ± 1.1	14.8	1904 ± 297	1342	-	-
**CTs-rich extract from *Alnus glutinosa* bark extracted by 50% EtOH (46% CTs on o.d. extract)**						
**CTs–PEI (10% of** **pinewood particles mass)**	5.2 ± 1.2	2.9	682 ± 226	255	-	-
**CTs–PEI (20% of pinewood particles mass)**	16.0 ± 0.6	14.9	1914 ± 231	1477	0.45 ± 0.04	0.40
**CTs-rich extract from *Alnus glutinosa* bark extracted by water (24% CTs on o.d. extract)**						
**CTs–PEI (10% of pinewood particles mass)**	4.8 ± 1.1	2.7	696 ± 227	267	-	-
**CTs–PEI (20% of pinewood particles mass)**	12.1 ± 0.8	10.6	1283 ± 216	875	-	-
**EN 312 P2 standard**	-	11	-	1800	-	0.40

**Table 4:** Properties of used adhesive systems and obtained plywood.

Adhesive Composition (*w:w*)	Viscosity, mPa·s, at 25 °C	MOE, N/mm^2^		Shear Strength, N/mm^2^		Density, kg/m^3^	Moisture, %
		Perpendicular to Wood Grains	Parallel to Wood Grains	After Immersion in Water at 20 °C (Class 1)	After Cyclic Treatment in Boiling Water (Class 2)		
**ULEFR**	810 ± 30	1190 ± 160	16,270 ± 1800	2.28 ± 0.38	1.68 ± 0.30	666	8–9
$L_{5\%}^{q}$	-	888	12,868	1.56	1.11	-	-
**(CTs–PEI):** **ULEFR = 2:8**	124,680 ± 1800	1000 ± 150	14,610 ± 660	1.79 ± 0.38	1.56 ± 0.28	688	8–9
$L_{5\%}^{q}$	-	717	13,362	1.07	1.03	-	-
**(CTs–PEI):** **ULEFR = 4:6**	>3,000,000	1070 ± 140	14,480 ± 880	1.84 ± 0.34	1.36 ± 0.27	722	8–9
$L_{5\%}^{q}$	-	805	12,817	1.20	0.85	-	-
**(CTs–PEI):** **ULEFR = 1:1**	>3,000,000	1030 ± 110	14,085 ± 1300	2.04 ± 0.29	1.30 ± 0.32	702	8–9
$L_{5\%}^{q}$	-	822	11,628	1.49	0.70	-	-
**(CTs–PEI):** **ULEFR = 6:4**	>3,000,000	1030 ± 120	13,980 ± 1730	1.45 ± 0.23	1.30 ± 0.29	644	8–9
$L_{5\%}^{q}$	-	803	10,710	1.02	0.75	-	-
**(CTs–PEI):** **ULEFR = 8:2**	>3,000,000	1060 ± 200	11,720 ± 1380	0	0	736	8–9
$L_{5\%}^{q}$	-	682	9112	-	-	-	-

**Table 5:** Influence of grey alder bark extraction residues applied as an additive to the adhesive system, on the mechanical properties of plywood.

Adhesive Composition (*w:w*)	Pretreatment of Plywood Sample	Shear Strength, N/mm^2^	$L_{5\%}^{q}\;$
**CTs–PEI-ULEFR (6:4)**	After immersion in water at 20 °C (Class 1)	1.45 ± 0.23	1.02
**CTs–PEI-ULEFR (6:4) + 10% filler**		1.62 ± 0.26	1.13
**CTs–PEI-ULEFR (4:6)**	After cyclic treatment in boiling water (Class 2)	1.36 ± 0.27	0.85
**CTs–PEI-ULEF (4:6) + 10% filler**		1.40 ± 0.18	1.06

## Data Availability

Data are contained within the article.
